# Major updates to FDA-recognized Clinical and Laboratory Standards Institute breakpoints are a win for the fight against antimicrobial resistance

**DOI:** 10.1128/jcm.00106-25

**Published:** 2025-04-14

**Authors:** Romney M. Humphries, Patricia J. Simner

**Affiliations:** 1 Department of Pathology, Microbiology and Immunology, Vanderbilt University Medical Center 12328, Nashville, Tennessee, USA; 2 Department of Pathology, Johns Hopkins University School of Medicine 1500, Baltimore, Maryland, USA; 3 Department of Medicine, Johns Hopkins University School of Medicine 1500, Baltimore, Maryland, USA; Boston Children's Hospital, Boston, Massachusetts, USA

## Abstract

Antimicrobial resistance (AMR) is a pressing crisis for global health. At the front lines of detecting AMR are clinical laboratories, which perform antimicrobial susceptibility testing (AST). In recent years, the ability of laboratories to conduct this testing in the United States has been challenged by changing interpretive standards and increased regulation surrounding laboratory testing, most recently U.S. Food and Drug Administration (FDA) regulation of laboratory-developed tests. In early 2025, the FDA recognized many breakpoints published by the Clinical Laboratory Standards Institute, including for microorganisms that represented an unmet need. This unprecedented step heralds a pragmatic approach to AST by the FDA and is a major win for laboratories, clinicians, and patients in the United States and globally. In this commentary, we discuss these changes and the impact on clinical laboratories.

## COMMENTARY

Antimicrobial resistance (AMR) affects 2.8 million Americans annually. At the forefront of detecting AMR is the clinical microbiology laboratory, which performs antimicrobial susceptibility testing (AST) among clinical and surveillance isolates of bacteria, mycobacteria, and fungi. In the United States, both manual and automated AST devices marketed for testing of human isolates require clearance by the U.S. Food and Drug Administration (FDA). Historically, when an FDA-cleared AST was not available for a given antimicrobial-organism combination, clinical laboratories performed testing off-label, using breakpoints published in Clinical and Laboratory Standards Institute (CLSI) standards and guidelines, a modification that rendered these “laboratory-developed tests” (LDT). Several major changes over the past two decades have made the task of performing AST increasingly complex for clinical laboratories, impacting the care of patients in the United States.

Starting in 2006, the FDA started to require the use of FDA-recognized susceptibility test interpretive criteria (STIC), also known as breakpoints, on FDA-cleared devices. Historically, the use of either CLSI or FDA breakpoints was accepted by the FDA (1). For many years, this distinction was not an issue, as (1) FDA and CLSI breakpoints were often aligned and (2) in cases where CLSI breakpoints existed without an FDA equivalent, AST devices could continue to be marketed with pre-2006 clearance. However, CLSI started making major revisions to breakpoints starting in 2010 (1). These revisions were in response to increasing AMR, novel AMR mechanisms, development of sophisticated pharmacokinetic/pharmacodynamic models for antimicrobials that better predict treatment response, and, importantly, publications that showed treatment failures for patients with “susceptible” isolates, all suggesting breakpoints were inaccurate (2, 3). In 2016, the 21st Century Cures Act went into effect, which enabled a mechanism for the FDA to recognize CLSI breakpoints and required review every 6 months (1). However, coordination of updates between CLSI and the FDA proved to be complex, and the FDA was often unable to recognize CLSI breakpoint updates (4). Reasons for nonrecognition included use of off-label doses of antimicrobials to inform susceptible breakpoints or lack of clinical data for a specific species (we refer the reader to FDA rationale documents found at https://www.fda.gov/drugs/development-resources/notices-updates). In 2024, there were over 100 differences between FDA and CLSI breakpoints, including many instances where the FDA did not recognize any breakpoint for antimicrobials in frequent clinical use (4). Delays for FDA recognition of CLSI breakpoints, compounded by delays for AST device companies obtaining clearance for their devices with these revised breakpoints, have led to a scenario where many laboratories continued to apply breakpoints that were more than 10 years out of date (5). AST devices are classified as class II, meaning updates, while encouraged, are not mandatory under FDA regulations. Furthermore, this situation led to legacy AST devices being placed at a disadvantage when updating their systems, as claims for antimicrobial agent-organism combinations not recognized by the FDA were lost (3). Also, newer technologies were limited in the number of antimicrobial agents and organisms that could be tested, making the introduction of these new technologies challenging for laboratories and minimizing the potential positive patient benefits associated with new test methods (4).

Nonetheless, disconnects between CLSI and FDA were manageable through the ability of clinical laboratories to validate AST devices for off-label use of CLSI breakpoints. This possibility was put into jeopardy following the FDA’s final rule on LDTs, which went into effect in 2024 (Laboratory Developed Tests | FDA). This rule clarifies that LDTs are *in vitro* diagnostic devices subject to FDA regulatory oversight and will phase out the FDA’s previous enforcement discretion policy. Examples of the types of AST impacted by the ruling include the following:

1. Modification of an FDA-cleared AST device to interpret results with current breakpoints. This may include updating to CLSI breakpoints, or current FDA breakpoints, if the device was cleared with historical, obsolete FDA breakpoints. For example, updating current ciprofloxacin and levofloxacin breakpoints for the *Enterobacterales* and *Pseudomonas aeruginosa* on an automated AST device that was FDA cleared using obsolete breakpoints.
2. Modification of an existing test for use with a new organism-antimicrobial agent combination for which the device is not cleared for use. For example, validation of a novel AST device for doxycycline and *Staphylococcus aureus*. The lack of FDA-recognized breakpoints precludes the ability for manufacturers, including laboratories, from obtaining FDA clearance for the combination.
3. Development of AST methodologies that are not considered reference methods (i.e., tests other than reference broth microdilution). For example, broth disk elution method for colistin and the combination of aztreonam plus ceftazidime-avibactam, as endorsed by CLSI, would constitute an LDT. The FDA recognizes the CLSI broth microdilution method as described in M07 as a reference method, used for device clearance; it is neither an LDT nor an *in vitro* diagnostic test (6).

A few areas of planned FDA enforcement discretion exist in the final rule. The most pertinent exception includes LDTs implemented by the laboratory prior to 6 May 2024 (when the rule went into effect) and those offered within an integrated healthcare system to meet an unmet medical need of patients receiving care within the same healthcare system. Application of updated clinical breakpoints that do not have FDA-cleared devices could be such an unmet clinical need. The issue with this latter exception is that many laboratories send testing to reference laboratories for these unmet needs. Reference laboratories serve patients outside of their healthcare system, and thus any AST LDTs offered after 6 May 2024 by these laboratories require FDA clearance prior to marketing (4). The impasse is that FDA clearance by the reference laboratory for these unmet needs is only possible if there are published FDA breakpoints for the organism-antimicrobial agent combination, making this testing impossible for reference laboratories. For many organisms, seeking new clearance and breakpoints via a new drug application pathway with the FDA is either impossible (due to rare occurrence) or implausible (due to high cost and lack of a sponsor). This exception may result in delays for reference laboratories or clinical laboratories from updating to current CLSI breakpoints in order to avoid submission to the FDA until such a time as commercial manufacturers (if one exists) make the update and seek FDA clearance. Furthermore, public health laboratories are subject to the FDA ruling, making the offering of important tests, such as ceftazidime-avibactam-aztreonam testing by the Antibiotic Resistance Laboratory Network, into jeopardy.

In January 2025, the FDA released major updates to the STIC website, which included recognition of many CLSI breakpoints by the FDA for the first time (i.e., most of the M45 breakpoints). Importantly, the FDA recognized the standards published in the CLSI M100 35th edition (aerobic and anaerobic bacteria), the CLSI M45 3rd Ed (infrequently isolated or fastidious bacteria), CLSI M24S 2nd Ed (mycobacteria, *Nocardia* spp., and other aerobic *Actinomycetes*), M43-A 1st Ed (human mycoplasmas), M27M44S 3rd Ed (yeast), and M38M51S 3rd Ed (filamentous fungi). Many of the breakpoints in these standards are recognized now for the first time by the FDA. It cannot be overemphasized what a major advancement for combating AMR and managing patients with complex infections these changes present. Many of the standards recognized are based on historical data and for microorganisms for which no clinical trial data or contemporary pharmacokinetic-pharmacodynamic studies are likely to be conducted. Nonetheless, for decades, these breakpoints have been used in the care of patients with serious infections caused by infrequently isolated microorganisms. Resistance has been reported for these organisms, and knowledge of the relative resistance of the bacterium is incredibly powerful data to aid clinicians in the practice of medicine (7). Furthermore, AST for these organisms feeds surveillance and public health efforts to address emerging infectious threats to patient populations. Recognition of these CLSI standards by the FDA enables AST by clinical laboratories and is a pragmatic solution to the management of the diverse microbes that cause infections among patients in the United States. This change provides a pathway for commercial manufacturers to develop tests for these pathogens, which fosters access globally for improved AST methods that may not have been good investments for companies historically, given the size of the American market (3).

**Fig 1:**
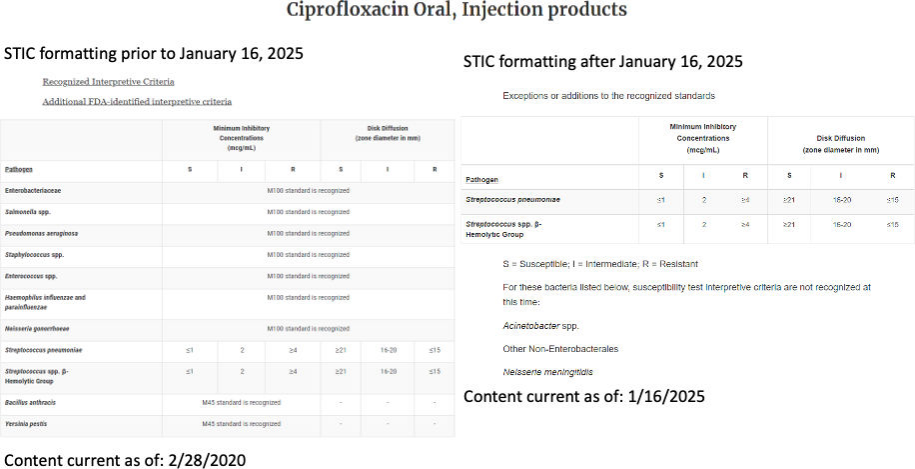
Updates to FDA STIC Website.

The recognized standards are listed both on the notice of updates and on each of the antibacterial (Antibacterial Susceptibility Test Interpretive Criteria | FDA) and antifungal (Antifungal Susceptibility Test Interpretive Criteria | FDA) STIC webpages, as appropriate. Significant changes have been made to the structure of the FDA’s STIC webpages. Instead of listing CLSI breakpoints that are recognized by FDA, now only exceptions or additions (i.e., where no CLSI breakpoints are available) are listed (Fig. 1). As highlighted in Fig. 1A for ciprofloxacin, prior to 16 January 2025, all CLSI recognized breakpoints were listed in addition to FDA-only breakpoints for *Streptococcus pneumoniae* and *Streptococcus* spp. β-hemolytic group. After 16 January 2025, only the additions and any exceptions from the recognized standards are listed (Fig. 1B). For example, the FDA does not recognize the CLSI ciprofloxacin breakpoints for *Acinetobacter* spp., the non-*Enterobacterales* and *Neisseria meningitidis* as published in the CLSI M100 34th edition, and these are listed as exceptions on the webpage (Fig. 1B). Thus, unless otherwise stated, the breakpoints published in any of the recognized standards are recognized by the FDA (Fig. 1B; e.g., ciprofloxacin for the *Enterobacterales*, *Pseudomonas aeruginosa*, *Staphylococcus* spp., and *Enterococcus* spp.).

The College of American Pathologists requires laboratories to make updates to AST breakpoints within 3 years of publication by the FDA (4). The best way to identify the date of recognition of a CLSI breakpoint by the FDA is to review the Notice of Updates, which includes breakpoints recognized between 28 February 2020 and 15 January 2025. Older updates to the STIC website can be found through review of changes to the federal register, but in most cases, this information should not be needed by the laboratory.

This positive change enables both routine AST by clinical laboratories and also development of novel AST devices. The change follows substantial effort and partnership between the FDA and CLSI and is a large step forward in the fight against AMR not only in the United States but also globally. However, navigating U.S. AST regulations remains complex. There remains significant uncertainty for the future of LDT regulation. As it stands, laboratories that practice in the United States and update testing performed by their laboratory to use CLSI breakpoints that are either not recognized by the FDA or have not yet been FDA-cleared on their device must list these as LDTs with the FDA. If this testing is to be done for patients outside the laboratory’s healthcare system, these updates may need FDA clearance, which would likely require use of FDA-recognized breakpoints. Ongoing work between the FDA and CLSI seeks to achieve recognition of high-priority CLSI breakpoints where the FDA has no breakpoint (e.g., staphylococcal breakpoints for trimethoprim-sulfamethoxazole or doxycycline). Many of these remaining breakpoints are “legacied” LDTs in most laboratories—i.e., in use before 2024. These pre-2024 breakpoints can and should continue to be applied by clinical laboratories. In addition, laboratories should keep up to date with FDA breakpoints at a minimum, a requirement for CAP-accredited laboratories (3). Importantly, when making changes to devices, laboratories must follow the instructions for use of the device, as the test conditions described in CLSI standards and guidelines are for reference methods (broth microdilution and agar dilution as described in M07 [8]) and the CLSI-standardized disk diffusion method as described in M02 (9). Modification of the testing conditions for commercial devices (e.g., adaptation of CLSI disk diffusion test conditions for gradient diffusion strips) is not validated by CLSI.

These challenges are complex. Clinical laboratorians should advocate for their patients, ensuring clinicians and policy makers are aware of the challenges of AMR and AST in the United States. Ongoing close collaboration between the FDA and CLSI is important, although governmental funding cuts may limit the FDA staff’s availability for this work. AMR is a healthcare crisis, and all parties must work with an eye to collaboration and compromise to ensure patients can be cared for optimally and safely in the United States and globally.
